# Degradation in carbon stocks near tropical forest edges

**DOI:** 10.1038/ncomms10158

**Published:** 2015-12-18

**Authors:** Rebecca Chaplin-Kramer, Ivan Ramler, Richard Sharp, Nick M. Haddad, James S. Gerber, Paul C. West, Lisa Mandle, Peder Engstrom, Alessandro Baccini, Sarah Sim, Carina Mueller, Henry King

**Affiliations:** 1 Natural Capital Project, Woods Institute for the Environment, Stanford University, 371 Serra Mall, Stanford, California 94305, USA; 2 Department of Mathematics, Computer Science and Statistics, St Lawrence University, 23 Romoda Drive, Canton, New York 13617, USA; 3 Department of Applied Ecology, David Clark Labs, North Carolina State University, 100 Brooks Avenue, Raleigh, North Carolina 27695-7617, USA; 4 Institute on the Environment (IonE), University of Minnesota, 1954 Buford Avenue, St Paul, Minnesota 55108, USA; 5 Woods Hole Research Center, 149 Woods Hole Road, Falmouth, Massachusetts 02540, USA; 6 Safety and Environmental Assurance Centre, Unilever R&D, Colworth Science Park, Sharnbrook, Bedfordshire MK44 1LQ, UK

## Abstract

Carbon stock estimates based on land cover type are critical for informing climate change assessment and landscape management, but field and theoretical evidence indicates that forest fragmentation reduces the amount of carbon stored at forest edges. Here, using remotely sensed pantropical biomass and land cover data sets, we estimate that biomass within the first 500 m of the forest edge is on average 25% lower than in forest interiors and that reductions of 10% extend to 1.5 km from the forest edge. These findings suggest that IPCC Tier 1 methods overestimate carbon stocks in tropical forests by nearly 10%. Proper accounting for degradation at forest edges will inform better landscape and forest management and policies, as well as the assessment of carbon stocks at landscape and national levels.

## Abstract (web summary)

Forest fragmentation is thought to reduce carbon storage at forest edges. Here, using remote sensing datasets, the authors show that biomass is 25% lower within 500 m of the forest edge, and suggest that fragmentation results in a global reduction in tropical forest carbon stocks by nearly 10%.

## Graphical abstract

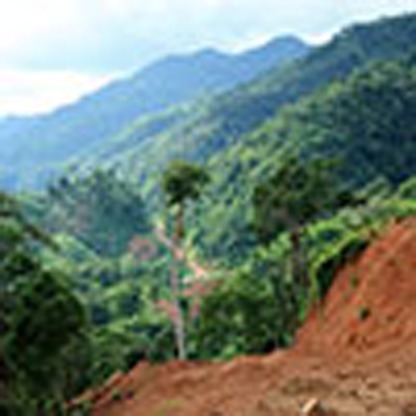

Forest clearing accounts for an estimated 12–15% of global greenhouse gas emissions^1^ through the annual loss of nearly 200,000 km^2^ of forest (an area about the size of Uruguay), a third of which is in the tropics^2^. These emissions are calculated through forest carbon inventories^3^,^4^, which do not take into account the decrease in carbon stocks that has been shown to occur in forests where they interface with converted land^5^. Experimental studies across Brazil have shown that biomass is reduced by between 9 and 50% within 100 m of the forest edge compared with the forest interior^6^,^7^. This is thought to be responsible for an estimated 600 Mg of carbon loss in the Amazon alone, and if extrapolated to the entire tropics, forest fragmentation could account for up to 24% of global carbon losses due to deforestation^8^. However, variation in the effect across different climates and habitats is poorly characterized. Considering that 70% of the world's forest area is within 1 km of the edge^9^, the extent to which this response is found across the tropics is of critical importance to carbon trading schemes and climate change mitigation more broadly.

Recent advances in remote sensing such as finer spatial resolution and improved algorithms for detecting biomass make it possible for the first time to assess landscape-level edge effects in forest carbon across the tropics. Here we calculate the effects of tropical forest fragmentation and increased area of forest edges on carbon stocks and document how widespread these effects are across the world. We illustrate the differences among regions, variability within regions, as well as factors that explain these differences. We show that ignoring edge effects can substantially overestimate carbon stored in fragmented forests, with implications for forest policy and management.

## Results

### The scale over which edge effects operate

**Figure 1:**
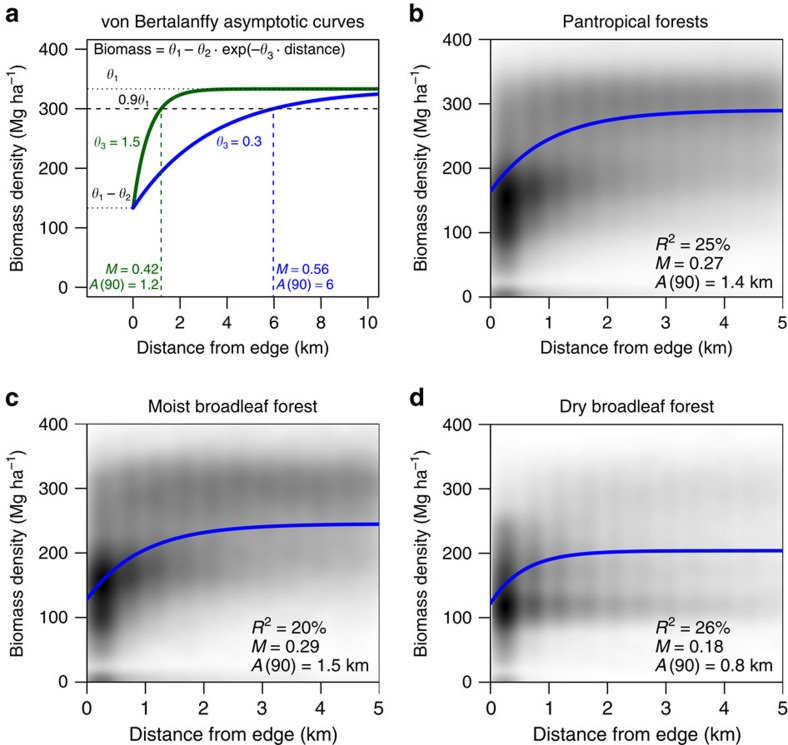
Edge effects on tropical forest carbon. Regression model for (**a**) demonstration purposes, at different scales, 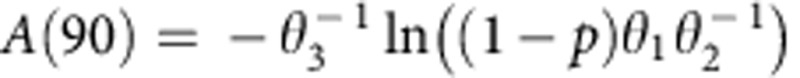, and for a given magnitude, *M*=*θ*_2_ exp(−0.232*θ*_3_)/*θ*_1_; (**b**) all pantropical forests; (**c**) moist broadleaf forests; and (**d**) dry broadleaf forests. Nonlinear least squares regression models were based on the entire set of pixels within the forest biomes, with a separate regression derived for each of the 10,000 km^2^ sub-regions (*N*=2,836). Plots show only a subset of the points (a random sample of over 1,000,000 pixels for the pantropics and 100,000 for each biome) to aid in display; grey shading in **b**–**d** denotes where the heaviest density of points lay. The curves in each plot are based on the model associated with the sub-region having the magnitude and scale closest to the weighted average of the whole pantropics (**b**), the moist broadleaf biome (**c**), or the dry broadleaf biome (**d**). The weighted averages for these regions are listed in the lower right of each panel.

We find that the scale of edge effects on forest biomass (defined as the distance between the forest edge and the point at which 90% of the asymptotic biomass is reached; *A*(90) in Fig. 1a) is an order of magnitude greater than is typically measured in field studies. We detect >10% reduction in above-ground biomass over scales of 1.5 km from the forest edge on average (Fig. 1b), far exceeding the 100–200 m range over which biomass has previously been modelled^8^ and over which both biomass and factors directly affecting biomass (for example, tree mortality, tree diameter, plant regeneration, canopy cover) have been measured in the field^5^,^10^. However, previously documented responses of other factors affecting biomass degradation near forest edges may explain our observed patterns. Forest edges affect microclimate (increasing wind speed to 400 m from the edge^11^), increase plant desiccation (up to 2.7 km from the edge^12^) and alter forest community structure and composition by increasing pioneer species (to 2 km (ref. 13)) and affecting phenology and recruitment (up to 5–10 km (ref. 14)). Anthropogenic fires in forests surrounding agricultural lands can penetrate up to 2.4 km (ref. 15), which may be exacerbated by other edge-related factors such as desiccation^12^. Taken together, the range of edge effects shown to influence the physical environment and plant communities support the extent of edge effects on biomass detected here.

### Magnitude of edge effects

Consistent with expectations from process-based modelling^16^, we estimate that biomass across the tropics is reduced an average of 25% within the first ∼500 m of the forest edge relative to forest interiors (Fig. 1b). This is several times greater than the declines documented empirically after fragmentation in average biomass (8.8% (ref. 6)) or large tree biomass (5–10% (ref. 17)) within 100 m of the forest edge, but less than the more extreme differences measured in the field between intact and degraded forest^7^. The magnitude of this edge effect (defined as the percent difference between the average biomass in forest edge pixels and the biomass predicted at the asymptote of the regression model; *M* in Fig. 1a) is on par with the differences in estimated carbon stocks among different forest types (for example, IPCC^3^ estimates 300 Mg ha^−1^ of above-ground biomass in American tropical rainforest compared with 220 Mg ha^−1^ in American tropical deciduous forest—a 25% difference). We therefore conclude that forest configuration can be as important to assigning values for carbon storage as differences among regions or ecosystems.

### Regional variation in tropical forest edge effects

**Figure 2:**
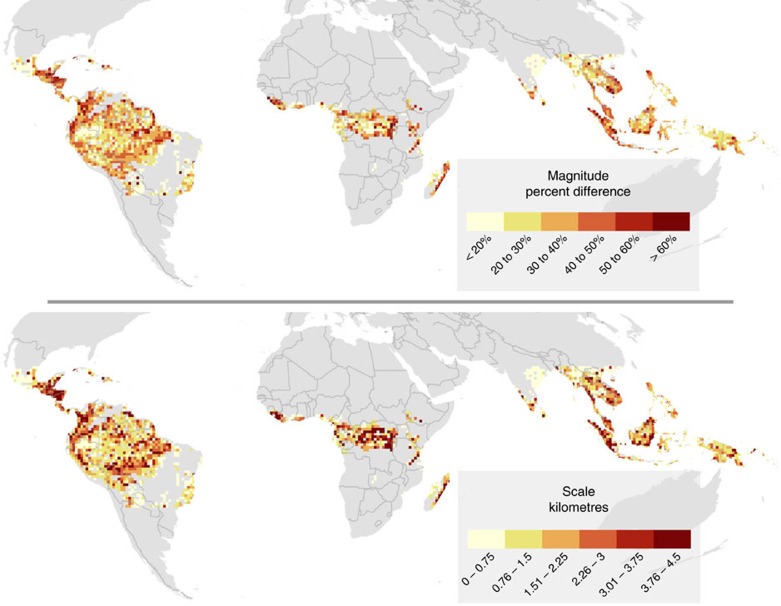
Magnitude and scale of forest carbon edge effects across the tropics. Edge effect relationships derived for tropical forest within each 10,000 km^2^ sub-region (*N*=2,836), with redder colours denoting stronger edge effects both in terms of magnitude of difference (% difference between biomass at forest edge and in forest interior) and scale of the edge effect (the distance from forest edge at which biomass is within 10% of the asymptotic biomass, or that seen in the interior of forests; in km).

The magnitude and scale of edge effects on forest biomass are consistently detectable across all continents, despite a large degree of variation among biomes (Fig. 1c,d) and regionally within biomes (Fig. 2). Edge effects in dry broadleaf forests are not as strong as those in moist broadleaf forests (18% versus 29%) and do not penetrate as far (0.8 versus 1.5 km). This fits with expectation, as dry forests should be less prone to edge-related desiccation than are moist forests, while moist forests tend to be denser than dry forests and thus should be more susceptible to edge-related wind turbulence^11^. At their strongest, edge effects can reach magnitudes of >60% and extend nearly 5 km into forest, such as across the moist forests of Veracruz, Mexico; Magdalena Valley, Colombia; the Albertine Rift in Africa; Madagascar; and the coastal swamps of Borneo and Sumatra (Fig. 2). *R*^2^ values for sub-region models vary between 0 and 0.83 (with a mean of 0.25) and larger *R*^2^ values generally correlate with higher magnitude and scale estimates^18^ (Supplementary Fig. 1).

The variation we observe in the magnitude and scale of edge effects in biomass can be explained in part by physical factors and human activity (Supplementary Table 1). Dry season length is negatively correlated with both magnitude and scale of edge effects, with up to 10% greater magnitude (in Africa) and extending >1 km further (in the Americas) for each month of a shorter dry season. Higher elevation is positively correlated with the magnitude and scale of edge effects in the Americas and Africa. Furthermore, the proportion of nearby working lands^19^, such as croplands, rangelands and populated forests, is associated with stronger edge effects, both in terms of the magnitude and the scale, across the tropics. These results correspond well with recent observations of tree density, which increases with elevation for tropical dry forests and with precipitation for tropical moist forests, and declines with human development in both forest types^20^.

## Discussion

The magnitude and scale of edge effects we report across the tropics can be used to specify a spatially explicit relationship between forest management and carbon stocks. This can help correct the discrepancy between widespread recognition of carbon edge effects in the scientific literature and their omission in current policy.

We find that assuming uniform biomass across forest patches equivalent to biomass levels found in forest interiors overestimates total carbon by nearly 20% in forest edge areas (extending 1.5 km into forests, on average). Accounting for edge effects would thus reduce the total carbon inventory across the tropics by 9.4 Pg. This is 30% to nearly three times greater than previous estimates of how much fragmentation compounds carbon losses over a 30-year period (that is, 0.11–0.24 Pg per year or 3.3–7.2 Pg over 30 years across the tropics^8^), but lower than suggested by empirical analyses of how much accounting for fragmentation can alter estimates of carbon emissions from deforestation^21^.

Greater precision in magnitude and scale of edge effects on biomass will be enabled by greater frequency and resolution of remotely sensed data products. Such advancements in data will allow for further analyses probing the intensity of edge effects over smaller spatial scales or over time. Even at the 500 m scale of currently available pantropical data, however, the magnitude and scale of edge effects on biomass averaged over that coarse of an area are large—and the consequences are substantial enough to suggest that some revision of current carbon policy is warranted.

National greenhouse gas inventories and other carbon accounting systems using Tier 1 IPCC methods^4^ assign a fixed carbon stock value by vegetation type without adjusting for edge effects, which likely underestimates the carbon loss due to forest fragmentation. Better accounting for edge effects in carbon stocks will improve forest and climate policies for a variety of applications: landscape zoning and forest management strategies for effective reforestation, restoration or conservation planning^22^; sector, corporate and product level environmental accounting^23^ and action (for example, zero deforestation commitments^24^,^25^, moratoria^26^); and emerging carbon markets or carbon trading programs like REDD+ to secure and enhance the climate regulation service provided by forests^27^. In these many decision contexts, a hectare of forest should not be viewed as exchangeable with just any other hectare of forest, because the value of that habitat for carbon storage depends upon the configuration of forest around it.

The increasing threats to tropical forests stemming from an expected population of 6 billion people in the tropics by 2,100, and the expansion of 200 million hectares of agriculture and construction of 25 million km of roads by 2,050 (ref. 28) underscore the urgency of understanding the full ramifications of forest fragmentation and degradation. The magnitude of edge effects demonstrated here should be given serious consideration to plan forest conservation and climate mitigation most effectively.

## Methods

### Analytic process and data sources

We quantify the reduction in carbon storage near forest edges using geospatial calculations^29^ on publicly available global data sets of pantropical data sets on above-ground biomass^30^, associated land cover^31^ and other factors (see ‘Modelling edge effect response to biophysical and human factors' section below), implemented by a Python-based computational pipeline^32^. The pipeline identifies 10,000 km^2^ sub-regions in forests across the tropics (*N*=2,836), calculates the distance from forest edge for every pixel within those sub-regions^33^, and aggregates relevant biophysical and human variables^34^ per pixel and per sub-region for further statistical analysis that results in maps of edge effects across the tropics^18^,^35^. Within each sub-region, we construct an asymptotic regression model between forest biomass and distance from forest edge for all of the forest pixels (see ‘Modelling biomass density via distance to forest edge' section below). Forest edge is defined as any forest pixel adjacent to a non-forest pixel; we measure the Euclidian distance of every forest pixel (at ∼500 m resolution) to the nearest edge. While variation may exist within the area of a pixel, this represents the finest scale globally available biomass data set. Furthermore, and importantly for the context of our study, if edge effects exist beyond 500 m, they will be detectable at this resolution.

The biomass data were created by the Woods Hole Research Center^30^ and the associated land cover map comes from the MODIS data set using the International Geosphere-Biosphere Programme (IGBP) classification^31^. This classification uses >60% tree cover exceeding 5 m height as a threshold for forest cover^36^. Woodlands are defined as 40–60% tree cover and wooded grasslands/savannas have between 10 and 40% tree cover (both exceeding 5 m height). This designation likely excludes any early successional or highly disturbed forests.

To calculate distance to forest edge, we constructed a raster map based on the MODIS land cover map whose original forest pixels were transformed into the shortest Euclidian distance from the current pixel to forest edge. Forest edge distances are calculated by first masking all global land cover MODIS layers that correspond to forest land cover IDs, (specifically, evergreen needleleaf forest (1), evergreen broadleaf forest (2), deciduous needleleaf forest (3), deciduous broadleaf forest (4) and mixed forest (5)). Distances from edge are calculated for each forest cell as the Euclidean distance from cell-centre to centre of the nearest non-forest cell using the Euclidean distance transform in the PyGeoprocessing Python library^29^. While variability can be expected in the true edge detected within a 465 m pixel, forest can be assumed to occupy more than half of the pixel defined as forest and less than half of any pixel not defined as forest; therefore the distances may be considered to extend ±232 m.

### Assumptions and mechanisms in the underlying data

The distance to forest edge was calculated using the MODIS land cover type product (MCD12Q1) with a 17-type classification developed by the International Geosphere-Biosphere Programme (IGBP). This product has been tested for errors using a 10-fold cross validation of the 1,860 sites contained in the training data set, which resulted in a reliability estimate of 72–77% for forest types 1–4, and 53% for type 5, mixed forest^31^. However, the vast majority of that error is between the different forest types; types 1, 2, 3 and 5 have a reliability of 93–98% for correctly identifying whether or not the land cover type is forest. Type 4, deciduous broadleaf forest, has a slightly lower reliability for correctly identifying forest (84%), being most commonly confused (12% of the time) with woody savanna. Error in identifying forest could lead to bias in the detection of the forest edge, but the deciduous broadleaf forests are poorly represented throughout the tropics relative to other forest types. They comprise the plurality of forest cover (>5% higher cover than the rest of the forest types) in only 2% of the regression model sub-regions, and thus cannot be driving the edge effects seen here.

The underlying biomass data used in this analysis were generated using LiDAR data from the Geoscience Laser Altimeter System (GLAS) trained with data of biomass measured on the ground in 283 sites across the tropics; the calculations for above-ground biomass are based on tree diameter and wood density (hereafter referred to as the Baccini method^30^). A similar method (hereafter referred to as Saatchi method^37^) added tree height as a third parameter in this calculation. The two-parameter equation in the Baccini method assumes a non-varying ratio of tree diameter to tree height, which has been shown to cause a 10–20% overestimate in total biomass; this bias is strongest in South America, where trees are shorter compared with global averages of height for trees of the same diameter^38^. However, it is unlikely that this bias would impact estimates of edge effects, since the height to width ratio in trees should not vary within a single forest fragment. Furthermore, although comparison of the two data sets on a pixel level showed substantial differences in biomass, the direction of the difference was consistent over the spatial extents relevant to the detection of edge effects^38^. A comparison of edge effects with the method using a three-parameter equation in the Saatchi method was impractical because of the coarser resolution of the data generated using this method (1 km for Saatchi compared with 500 m for Baccini). The scale of our analysis also integrates over some within-pixel variation inherent in MODIS products, although the Baccini method minimizes much of the reflectance error arising from adjacent pixels^39^. Overall, biomass estimated by the Baccini method accounts for 75% of the variation in the GLAS LiDAR estimates for biomass across the tropics, and has a root mean squared error of 25, 19 and 24 Mg C ha^−1^ for tropical America, Africa and Asia, respectively^30^.

The remote-sensing methods for calculating biomass using the Baccini method are consistent with the physical mechanisms associated with the variation in forest edges observed here. First and perhaps most obviously detected by remote-sensing, canopy cover is directly linked to biomass, and reductions in canopy cover from thinning or degradation can be seen in forest edges. Second, the age structure in the forest edges is characterized by younger and thinner trees. This leads to lower biomass overall, detected in remote-sensing through the effect of shadow amount in the reflectance values in short-wave infrared wavelength region (for example, bands 6 and 7 of MODIS^40^). In this wavelength region, the amount of diffuse radiation is limited and shadows play a significant role, demarcating the forest structure. Finally, the short-wave infrared bands are also sensitive to water content in the vegetation, an indicator of general tree health, which is compromised by pests, disease and desiccation, all shown to be more common in forest edges. Some biases in biomass have been identified^30^ for example, biomass tends to be overestimated in dry sparse forest. This may partially account for the generally stronger edge effects seen in moist forest, as compared to dry forest, but does not undermine the existence of the pattern.

It is important to note that by utilizing remote-sensing data, our approach accounts for above-ground biomass only, and thus does not include considerations for how below-ground or soil carbon may be impacted by edge effects. This makes it a less reliable estimate in regions where soil carbon makes up a large proportion of total carbon stock, such as for peat soils in Southeast Asia.

### Modelling biomass density via distance to forest edge

We model the relationship between biomass density (Mg ha^−1^) and distance to forest edge (km) using a von Bertalanffy asymptotic regression model^41^ of the form *b*(*d*)=*θ*_1_−*θ*_2_ exp(−*dθ*_3_), where *b* is biomass, *d* is distance to closest forest edge, *θ*_1_ is the asymptotic biomass (the point at which the edge no longer has any effect), *θ*_1_−*θ*_2_ is the average density at the theoretical forest edge (that is, distance of 0 km), and *θ*_3_ controls the rate at which the asymptote is reached^18^. The nonlinear models were based on the entire set of pixels within the forest biomes and fit using the nls function in R (ref. 42). These relationships are plotted using the smoothScatter function of R based on a random sample of over 1,000,000 pixels for the pantropics and 100,000 for each biome (Fig. 1). Representative asymptotic regression curves were added to these plots based on the model associated with the 10,000 km^2^ sub-region having the magnitude and scale closest to the weighted average of the respective region (that is, the pantropics or biome associated with each plot).

Two different characteristics of the edge effect were then determined: magnitude and scale. The magnitude (*M*) is defined as the ratio of the biomass density at the forest edge relative to the biomass density in the forest core (*θ*_1_) and represents the proportional reduction in biomass density when moving from the core to the edge of the forest; specifically, *M*=*θ*_2_ exp(−0.232*θ*_3_)/*θ*_1_. Magnitude is bounded by 0 and 1, with strongest edge effects being closer to 1 and the majority of the data falling below 0.6 (Supplementary Fig. 1a). The scale of the edge effect was considered to be the distance (in km) where the average biomass equals *p*% of the estimated asymptote. It is calculated as 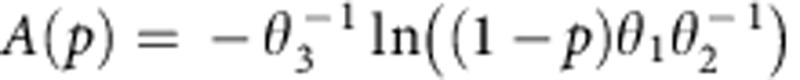 and is bounded by 0 and the maximum observed distance; the majority of the data fell below 2 km (Supplementary Fig. 1b). For the purposes of our study, we used a threshold of 90% of the estimated asymptotic biomass for this distance, but any value could be used, and we explored other nearby values, which generally displayed similar trends. To interpret this, a scale of *A*(90)=2 implies that at 2 km from the forest edge, the average biomass density has reached 90% of the asymptotic density (*θ*_1_).

### Modelling edge effect response to human and physical factors

For each 10,000 km^2^ sub-region throughout the tropics, we regressed magnitude and scale of edge effect against other physical and human factors, to investigate potential mechanisms behind the variation in severity of edge effect. The variables included were those hypothesized to have an impact on forest biomass and possible physiological response to fragmentation, and that also were readily available with global coverage.

Physical factors as predictor variables included: latitude, elevation^43^, precipitation^44^, soil water capacity^45^ and length of dry season. Dry season was estimated by stacking 12 months of precipitation rasters^44^ and for each pixel stack, counting the number of precipitation pixels that contained <60 mm of precipitation^32^. The result is a raster of the same dimensionality as the original 12 month data set whose pixel values contain this approximation of the dry season length in months.

For human factors, we first included the Intact Forest Landscape layer^46^ (IFL), which combines many aspects of human influence (for example, roads, transmission lines, other infrastructure, human settlements, anthropogenic fires and so on) and delineates the areas of forest that are free from all human influence for at least 500 km^2^. About half of the 10,000 km^2^ sub-regions intersect with the IFL layer; magnitude of edge effect is very similar within the intact (overlapping IFL) and non-intact forest landscapes, but scales over which edge effects operate are much greater in the intact landscapes (Supplementary Table 2).

We do not test for human influence within the intact forest (because by definition, these cells do not overlap with populated areas), but restrict the predictors of edge effect magnitude and scale to the physical variables outlined above. For the non-intact forests (that is, sub-regions in which the proportion of IFL=0), we used the following human factors as predictor variables: human population density^47^, livestock density^48^, fire intensity^49^, lighted area luminosity^50^ and the proportion of the sub-region covered by different anthromes^19^. Anthromes are spatially delineated areas describing the different types and intensities of human settlement, ranging from remote forests to working landscapes like agriculture and rangelands, to dense settlements.

Regression models were fit using robust linear methods (via the rlm function in R) using Tukey Bisquare weights to reduce the impact of outliers. Variable selection was guided by an exhaustive search in the regsubsets function from the leaps package in R (ref. 51) based on the Tukey Bisquare weights obtained from the full model. Additional fine tuning of the model, including obtaining weights specific for the reduced model, was performed using Bayesian information criterion (BIC) as a guide. For the intact forest (IFL>0) models containing a quadratic term, 95% bias-correction adjusted bootstrap confidence intervals (with 5,000 replicates as implemented through the boot function in R) are used to estimate the vertex of the quadratic curve and the corresponding response value. In the non-intact forest (IFL=0) models, only anthrome variables that had at least 10% non-zero values were considered as candidate explanatory variables. *R*^2^ values for robust linear models were approximated based on the weighted response variables (that is, magnitude or *A*(90)) and can be interpreted as the percent of variation in the weighted response that can be accounted for by the model. These outputs have been archived as a table and geospatial rasters that can be downloaded^18^ or explored through a web-app^35^.

### Calculating total carbon in forest edges

To estimate the overall reduction in carbon stocks due to edge effects, we calculated the sum of the biomass predicted by our regression model within each 10,000 km^2^ sub-region and compare it with the expected biomass if all forest pixels in the region contained the biomass density found in the interior of the forest (‘interior biomass' being defined as 90% of the asymptote). To convert biomass to carbon stock, we multiplied the biomass by 0.47, the IPCC carbon fraction for tropical forest^3^. We summed the total above-ground carbon present in forest edges in three ways. (1) To estimate total carbon predicted by our model, we calculated carbon using the regression model derived for each 10,000 km^2^ sub-region, for each forest pixel according to its distance from forest edge, and summed across all pixels in the *A*(90) edge areas (defined as above; the forest pixels within the distance from forest edge where modelled biomass is <90% of the asymptotic biomass)^18^. We found the modelled carbon stock within the edge areas to total 1.912 Mg C ha^−1^ × 21.466 ha per pixel × 10^9^ pixels, or 4.10 Pg of carbon. (2) To estimate the total biomass predicted by an assumption that all forest pixels contain the same biomass density as found in forest interiors, we multiplied the number of pixels in the edge areas by 90% of the asymptotic biomass. The carbon stock within edge areas according to this method totaled 2.349 Mg C ha^−1^ × 21.466 ha per pixel × 10^9^ pixels, or 5.04 Pg of carbon. (3) To check our predictions, we also compared the modelled values to the empirical values for biomass found in each sub-region. We found empirical biomass in the edge areas to total 1.847 Mg C ha^−1^ × 21.466 ha per pixel × 10^9^ pixels, or 3.96 Pg of carbon. When compared to the 4.10 Pg predicted by the regression model for these edge areas, these empirical values suggest the model tends to overestimate biomass by 3.5%.

To determine possible sensitivity to discretization effects, we explored the importance of the ‘missing biomass' effect (that is, a comparison of estimates assuming uniform or edge-related effects on biomass) as a function of distance to edge. We find that 33% (Supplementary Fig. 2) of tropical forests are within the *A*(90) edge areas (extending an average of 1.5 km into forests). Of those *A*(90) edge areas, another 30% are directly adjacent to non-forest in the MODIS land cover data set, and therefore within 465±232 m of the edge. However, the missing biomass effect extends multiple pixels away from the edge (Supplementary Fig. 3), thus is not driven exclusively by an artifact of the nearest to edge pixels.

### Data availability

Online access for all data and code included in these analyses are listed in refs 20 and 31-35

## Additional information

**How to cite this article:** Chaplin-Kramer, R. *et al.* Degradation in carbon stocks near tropical forest edges. *Nat. Commun.* 6:10158 doi: 10.1038/ncomms10158 (2015).

## Supplementary Material

Supplementary Information Supplementary Figures 1-3 and Supplementary Tables 1-2
