# Supplementary material for: Degradation in carbon stocks near tropical forest edges
Source: Nat Commun. 2015 Dec 18;6:10158. doi: 10.1038/ncomms10158 (PMC4703854; doi:10.1038/ncomms10158)
Supplement: Supplementary Information — Supplementary Figures 1-3 and Supplementary Tables 1-2 [file ncomms10158-s1.pdf]

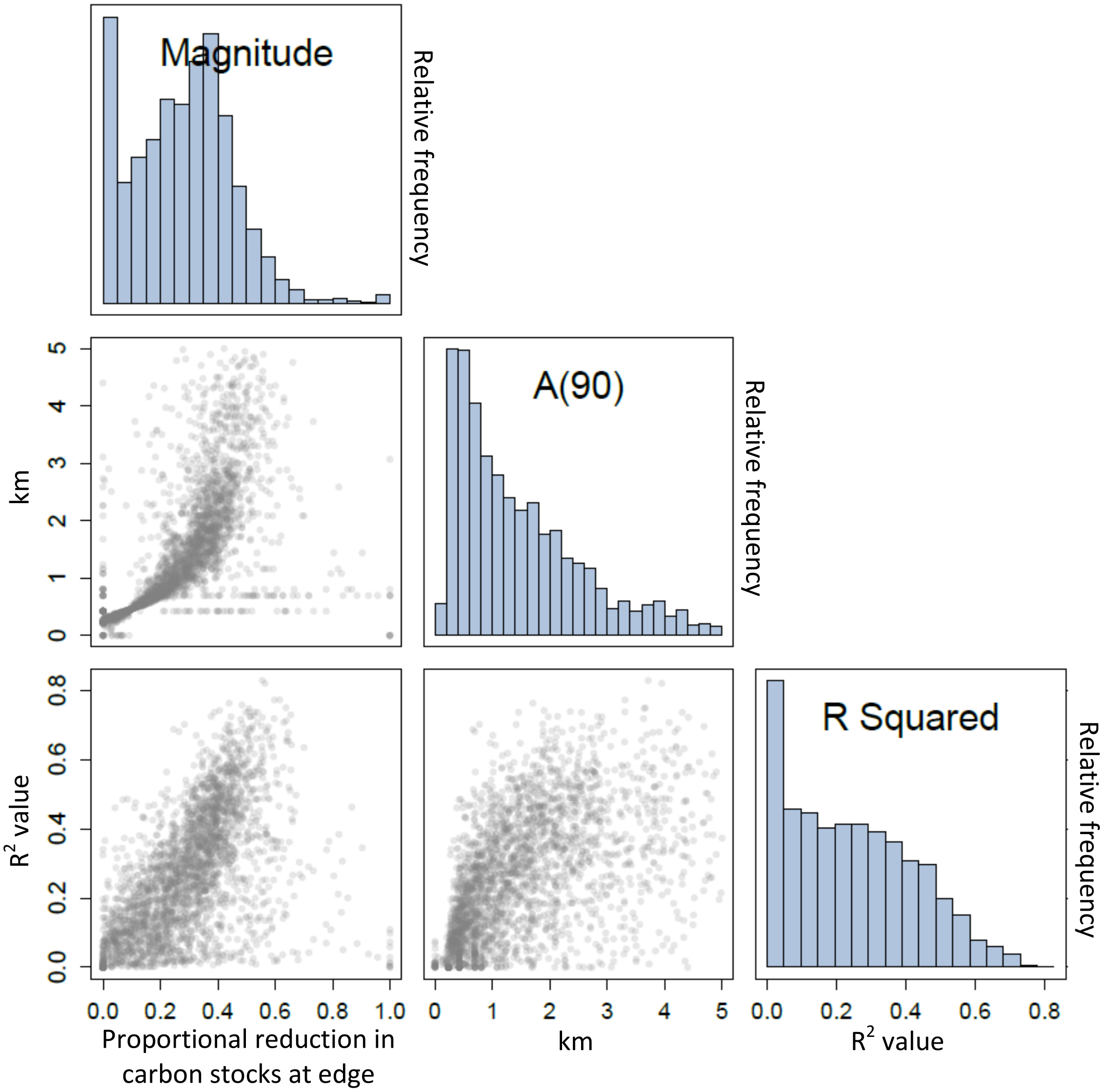

Supplementary Figure 1. Distributions of and relationships between magnitude, scale, and goodness-of-fit in the 10,000 km^2 sub-regions. Along the diagonal are histograms of magnitude of tropical forest carbon edge effects (defined as the proportional reduction in carbon between the forest edge and forest interior; see Methods), scale of tropical forest carbon edge effect (*A*(90), defined as the distance at which the modeled biomass is 90% of the asymptotic biomass according to the regression model; see Methods), and goodness-of-fit (R^2) of the edge effects models. The off diagonals provide bivariate plots between magnitude, scale, and good-of-fit of edge effect models.

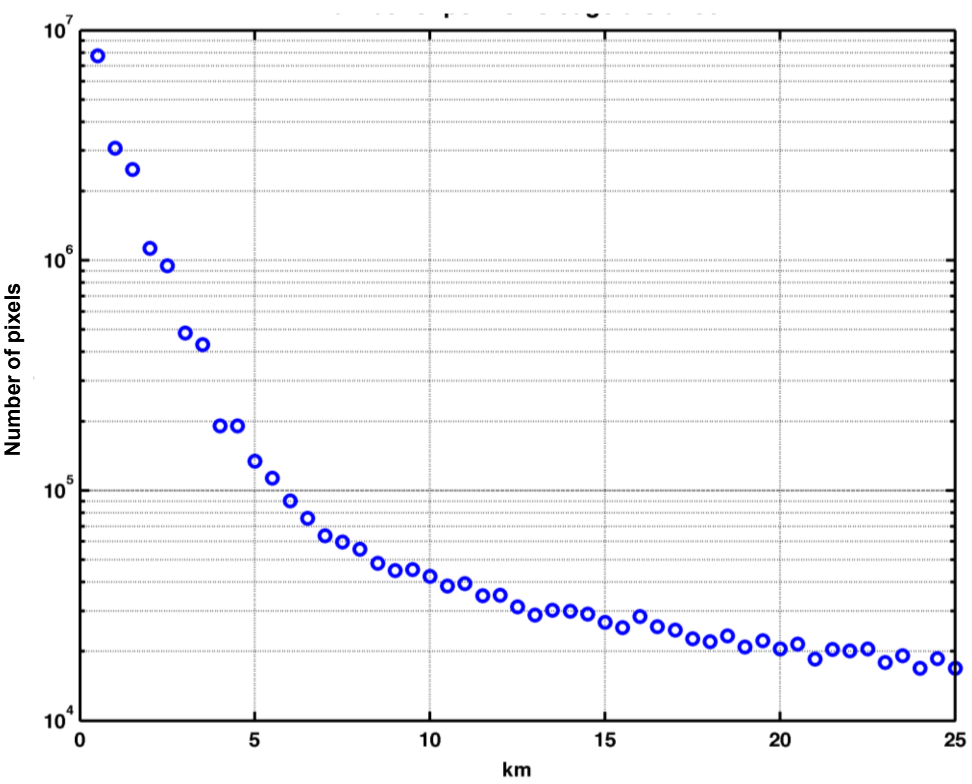

Supplementary Figure 2. Number of pixels at different distances from forest edge. Pixels are binned into 500 m segments centered at 500 m, 1000 m, 1500 m, etc. Only pixels falling within the A90 edge zone within their sub-region are shown here. Of the 60,477,382 forest pixels, we discarded those that had a zero biomass value, which inspection with Google Earth images indicated to correspond with water, leaving 56,703,961 forest pixels. Within that, 19,103,341 (33.7%) pixels fall within the edge region. Of those 19,103,341 edge pixels, 30.6% are edge-adjacent and an additional 10.0 % of all pixels are edge-diagonal.

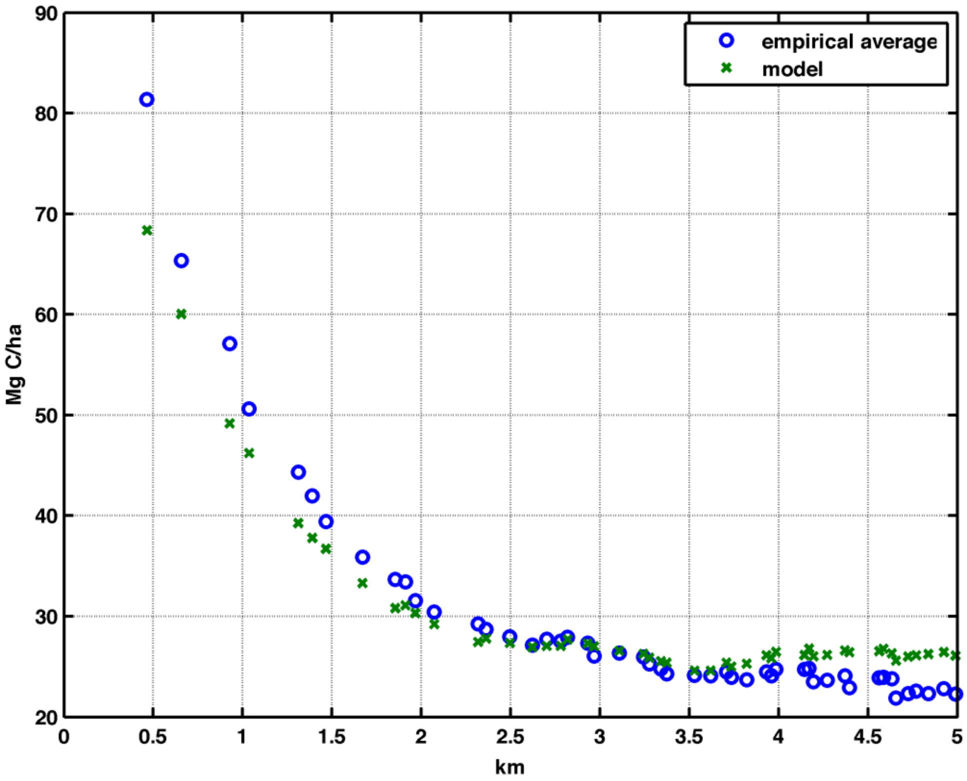

Supplementary Figure 3. Average missing biomass as a function of distance from forest edge. "Missing biomass" is defined at each pixel as the difference between 90% of the asymptotic biomass (derived from the regression for each 100 km x 100 km sub-region) and the pixel biomass (modeled or empirically-derived from the biomass dataset for each 500 m x 500 m pixel.) For the blue "o" curve, the pixel biomass is the satellite-based observation (Baccini et al. 2012). For the green "x" curve, the pixel biomass is taken as the biomass predicted by the von Bertalanffy model (Methods) fitted for the sub-region. While the von Bertalanffy model overpredicts missing biomass for distances less than ~2.5 km, this is a fairly small effect.

Magnitude (% difference)	Moist						Dry					
**Factor**	**Africa**		**Americas**		**Asia**		**Africa**		**Americas**		**Asia**	
	**N-I**	**IFL**	**N-I**	**IFL**	**N-I**	**IFL**	**N-I**	**IFL**	**N-I**	**IFL**	**N-I**	**IFL**
dry season (per month)	-3.3	0	-1.5	0.77	-2.5	3.7	-11	0	-1.6	0	-4	0
elevation (per 100 m)	0.84	2.69	0.75	0.3	0	0	3.68	0	0.56	2.6	0	0
precipitation (per 100 mm)	0	0.65	0	0.67	0.52	0.7	3.6	0	0	0	1.43	0
dense settlements	0	0	0	0	0.42	0	0	0	0	0	1.09	0
pastoral rainfed villages	0	0	0	0	0	0	0	0	0	0	0	0
rainfed mosaic villages	0	0	0	0	0	0	0	0	0	0	0	0
residential irrigated cropland	0	0	0	0	0.25	0	0	0	0	0	0	0
residential rainfed mosaic	0.21	0	0	0	0	0	0	0	0	0	0	0
populated rainfed croplands	0	0	0	0	0.54	0	0	0	0	0	1.65	0
residential rangelands	0	0	0	0	0	0	0	0	0	0	0	0
populated rangelands	0	0	0	0	0	0	0.85	0	0	0	0	0
populated forests	0	0	0.22	0	0	0	0	0	0	0	0	0
dry season ^ 2	0	0	0	0	0	-0.7	0	0	0	0	0	0
precipitation ^ 2	0	0	0	-0	0	-0	0	0	0	0	0	0
Model r-squared	***21%***	***31%***	***13%***	***10%***	***22%***	***11%***	***48%***	***-***	***7%***	***26%***	***47%***	***-***

Scale (meters)	Moist						Dry					
**Factor**	**Africa**		**Americas**		**Asia**		**Africa**		**Americas**		**Asia**	
	**N-I**	**IFL**	**N-I**	**IFL**	**N-I**	**IFL**	**N-I**	**IFL**	**N-I**	**IFL**	**N-I**	**IFL**
dry season (per month)	-85	-285	-83	362	-130	0	0	0	-31	-1230	-101	0
elevation (per 100 m)	0	230	0	60.3	0	0	-28	0	0	120	0	0
precipitation (per 100 mm)	0	0	0	0	3.29	7.3	0	0	3	16	4.7	0
dense settlements	0	0	0	0	22.4	0	0	0	0	0	28.5	0
pastoral rainfed villages	0	0	50.2	0	0	0	0	0	0	0	0	0
rainfed mosaic villages	22.8	0	0	0	0	0	0	0	0	0	0	0
residential irrigated cropland	0	0	66.9	0	9.9	0	0	0	0	0	0	0
residential rainfed mosaic	10.3	0	4.3	0	0	0	0	0	0	0	0	0
populated rainfed croplands	0	0	0	0	47.1	0	0	0	0	0	113	0
residential rangelands	0	0	0	0	0	0	9.7	0	0	0	0	0
populated rangelands	0	0	0	0	0	0	7.9	0	0	0	0	0
populated forests	8.2	0	24	0	15.4	0	0	0	0	0	0	0
dry season ^ 2	0	0	0	-46	0	0	0	0	0	122	0	0
elevation ^ 2	0	0	0	-0	0	0	0	0	0	0	0	0
precipitation ^ 2	0	0	0	0	0	-0	0	0	0	0	0	0
Model r-squared	***27%***	***9%***	***24%***	***7%***	***44%***	***11%***	***58%***	***-***	***10%***	***49%***	***57%***	***-***
Observations	***225***	***226***	***326***	***726***	***623***	***327***	***26***	***0***	***100***	***47***	***147***	***7***

Supplementary Table 1. Regression coefficients for predictors of edge effect magnitude and scale. Coefficients are listed as the amount of additional percentage difference in magnitude or additional meters of distance in the scale of edge effect, per each unit of predictor variable (i.e., per additional month of dry season, per 100 m gain of elevation, per 100 mm additional annual precipitation, or for each additional percentage point of any of the human land-uses). Zeroes denote non-significance. Each column represents a different statistical model, for moist and dry broadleaf forest on each continent, for non-intact forest (N-I) and for intact forest landscapes (IFL).

Magnitude	Africa				Americas				Asia			
	DBF		MBF		DBF		MBF		DBF		MBF	
	N-I	IFL	N-I	IFL	N-I	IFL	N-I	IFL	N-I	IFL	N-I	IFL
n	26	0	225	226	100	47	326	726	147	7	623	327
Mean	-0.19		-0.25	-0.3	-0.19	-0.12	-0.29	-0.33	-0.17	-0.28	-0.24	-0.31
St. Dev	0.11		0.26	0.16	0.22	0.16	0.18	0.13	0.26	0.24	0.21	0.13
Weighted mean	0.19		0.28		0.17		0.32		0.18		0.26	

Scale	Africa				Americas				Asia			
	DBF		MBF		DBF		MBF		DBF		MBF	
	N-I	IFL	N-I	IFL	N-I	IFL	N-I	IFL	N-I	IFL	N-I	IFL
n	26	0	225	226	100	47	326	726	147	7	623	327
Mean	0.63		1.03	2.56	0.72	0.94	1.16	1.80	0.79	1.55	1.01	1.74
St. Dev	0.275		1.012	2.504	0.513	1.323	0.849	1.299	0.833	0.903	0.87	1.109
Weighted mean	0.63		1.79		0.79		1.60		0.83		1.26	

Supplementary Table 2. Edge effects by continent and biome. Mean and standard deviation in magnitude (percent difference) and scale (km) of edge effects in tropical forests for different continents and biomes (dry broadleaf forest, DBF; moist broadleaf forest; MBF). Results are reported separately for non-intact forest (N-I) and intact forest landscapes (IFL), as well as a weighted mean across the two.
